# Suspected Calvarial Hyperostosis Syndrome Causing Different Ophthalmological Signs in Two Young Labrador Retrievers—Case Report

**DOI:** 10.1111/vop.70007

**Published:** 2025-02-23

**Authors:** Andrea Steinmetz, Stefan Kohl

**Affiliations:** ^1^ Department of Small Animals, Veterinary faculty University of Leipzig Leipzig Germany; ^2^ Tierärzte IVC Evidensia GmbH, Tierklinik Hofheim Hofheim Germany

**Keywords:** calvarial hyperostosis syndrome, computed tomography, dacryocystorhinography, epiphora, exophthalmos, nasolacrimal duct

## Abstract

**Objective:**

To describe calvarial hyperostosis syndrome (CHS) as a potential and unusual cause of exophthalmos or epiphora in young dogs.

**Animals Studied:**

A nine‐month‐old female intact (case 1) and a two‐year‐old male intact Labrador Retriever (case 2).

**Procedures:**

Patient history, including previous treatments, was documented. Both cases underwent physical and ophthalmological examinations, computed Tomography (CT), and histopathological analysis. Additional dacryocystorhinography (DCR) was performed in case 2.

**Results:**

Lateral exophthalmos of the left eye was the primary clinical sign in case 1. In case 2, serous lacrimal discharge was observed without other signs of ocular irritation. A slightly thickened and firm area distal to the medial canthus of the right eye was also noted. In this case, Jones 1 test on the right side was negative, whereas Jones test 2 revealed increased resistance when flushing the nasolacrimal system. CT imaging in both cases identified solid, smoothly marginated new bone formation. In case 1, this involved the left frontal bone, occipital bone, parietal bone, and temporal bone. In case 2, the new bone formation affected the right lacrimal bone and frontal process of the maxillary bone, leading to encasement and narrowing of the nasolacrimal duct (NLD). Histopathological analysis revealed active bone remodeling with osteoblasts in case 1, while case 2 demonstrated regularly differentiated trabecular lamellar bone with intertrabecular spaces. No evidence of neoplasia or osteomyelitis was observed in either case. Taken together, these findings strongly support a diagnosis of CHS in both cases.

**Conclusions:**

CHS involving flat bones can present as exophthalmos or epiphora and should be considered in the differential diagnoses of these conditions, particularly in young dogs.

## 1 Introduction

Calvarial Hyperostosis Syndrome (CHS) is a non‐neoplastic proliferation of the flat bones of the skull and has been reported in various animal species, including captive lions [1, 2], a cat [3], and dogs. In dogs, CHS was first described in Bullmastiffs [4, 5] and a Mastiff [6]. Subsequently, CHS was reported in other breeds, including Beagles [7], Pitbull Terriers [8, 9], Springer Spaniels [10], Weimaraners [11], mixed breeds [12] and Dalmatians [13].

In most cases, the affected bones included flat bones such as the occipital, frontal, parietal, and temporal bones. At the time of diagnosis, all dogs in these reports were between 4 and 9 months old. Common clinical signs included skull swellings, seizures, fever, and pain.

This report presents two cases of suspected CHS in young Labrador Retrievers, highlighting their distinct clinical and radiological features.

## 2 Case Report

### 2.1 Patient History

#### 2.1.1 Case 1

A nine‐month‐old intact female Labrador Retriever was referred to our clinic due to lateral exophthalmos of the left eye. The dog had previously undergone two surgical procedures at the referring veterinarian to address the left‐sided prolapsed gland of the third eyelid (TE), performed 3 and 2 months prior to presentation. Following the second surgery, the left eye appeared mildly reddened, with progressive exophthalmos noted over time. No signs of trauma or inflammation in the periocular region were reported.

#### 2.1.2 Case 2

A two‐year‐old male intact Labrador Retriever was referred to our clinic due to chronic serous discharge from the right eye, persisting for over 1 year. No other signs of ocular irritation, trauma, or periocular inflammation had been observed. The right eye had previously been treated by the referring veterinarian with dexamethasone‐chloramphenicol eyedrops (Cefenidex, CP Pharma), but the treatment provided only temporary relief without lasting improvement.

#### 2.1.3 Clinical examinations and results

**FIGURE 1:**
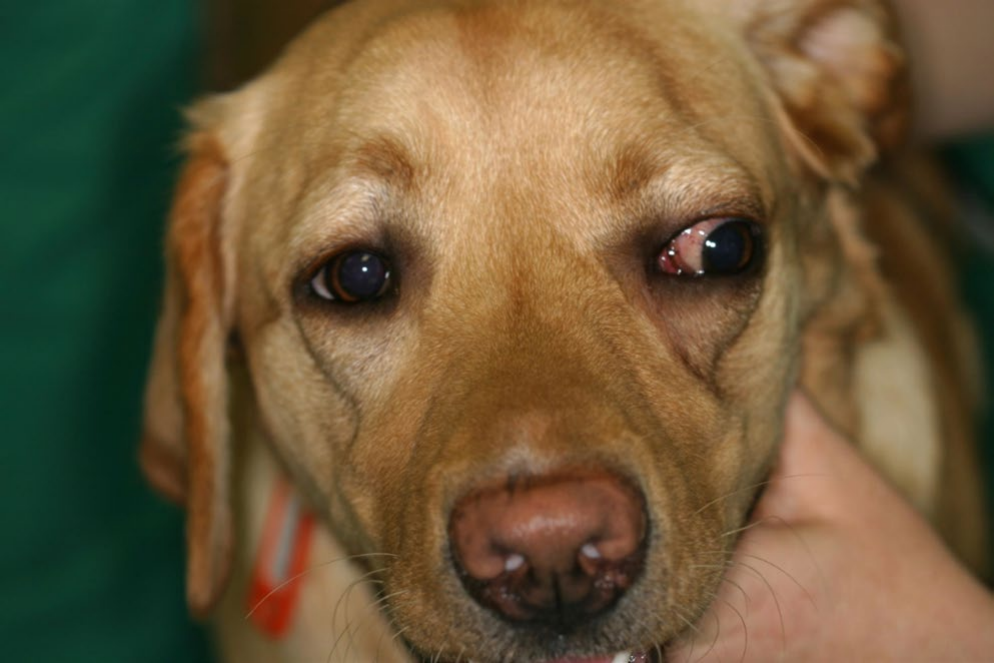
Case 1: Lateral exophthalmos of the left eye.

In case 1, the dog presented with lateral exophthalmos of the left eye (Figure 1).

**FIGURE 2:**
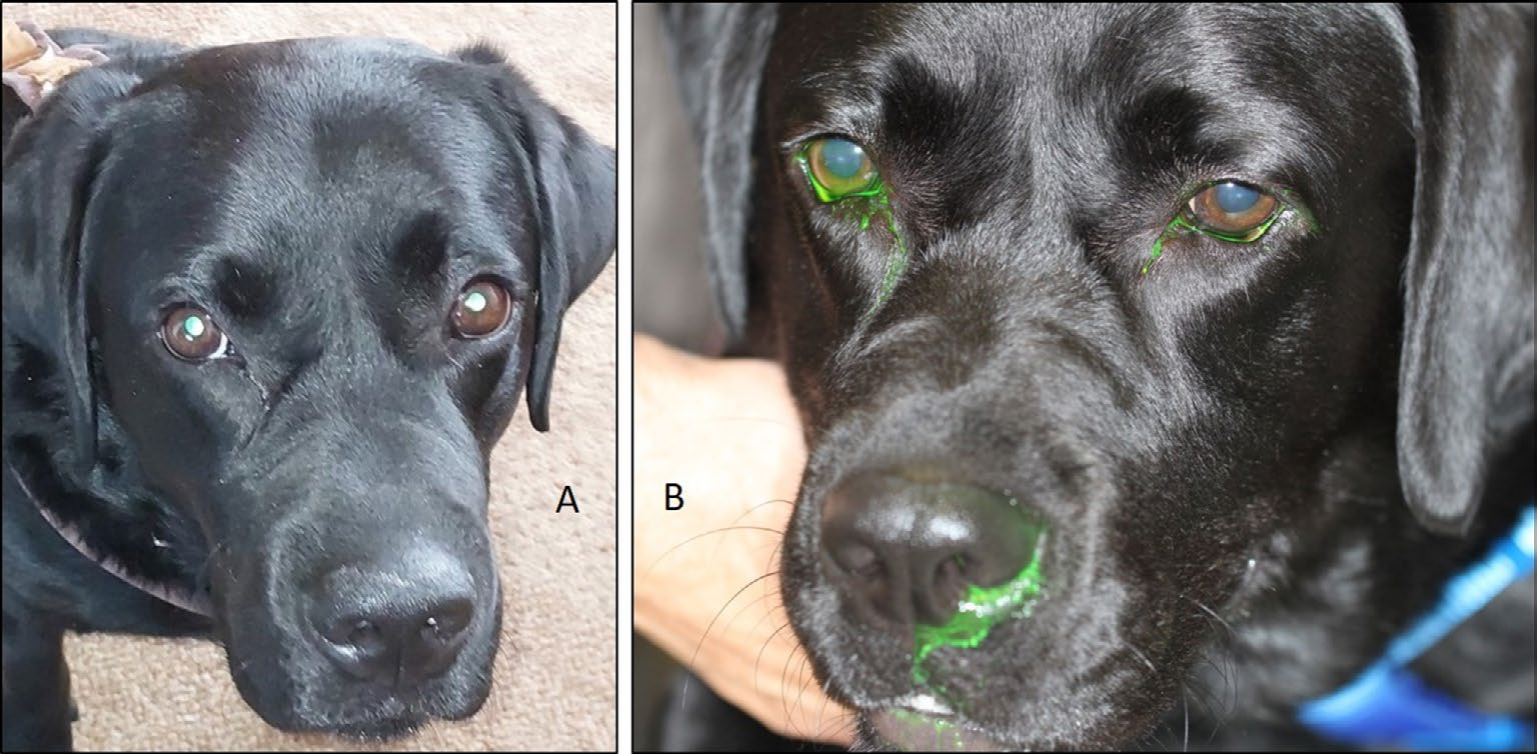
Case 2: The area distal to the medial canthus of the right eye appeared slightly thickened compared to the contralateral side (A) and showed a negative Jones 1 test on the right side (B).

Retropulsion of the globe was notably reduced, particularly in the dorsomedial direction. Palpation of the left skull and mouth opening elicited signs of pain. Neurological examination of both eyes showed no abnormalities. In case 2, a persistent serous discharge was observed from the right eye. The area distal to the medial canthus of the right eye appeared slightly thickened compared to the contralateral side (Figure 2A). Upon palpation, the area was firm but not painful. Neurological examination of both eyes was normal.

Slit‐lamp (SL 19, Kowa Company Ltd.) examination in both cases revealed conjunctival redness in the left eye of case 1 as the only significant finding. No abnormalities were identified in the anterior or posterior segments of either dog's eyes.

In case 2, Jones test 1 and 2 were performed. On the left side, both tests were positive. On the right side, Jones test 1 was negative (Figure 2B), while Jones test 2 indicated increased resistance compared to the left side.

Otherwise, physical examinations of the patients revealed no significant abnormalities.

Based on the clinical and ophthalmologic findings, computed Tomography (CT) of the head was indicated for both cases, with an additional dacryocystorhinography (DCR) conducted in case 2.

#### 2.1.4 Further Diagnostics and Results

Anesthesia was induced intravenously with 1 mg/kg ketamine (Ketamin 10%, WDT), 6 μg/kg medetomidine hydrochloride (Domitor, Vetoquinol), 0.5 mg/kg diazepam (Solupam, Dechra), 4 mg/kg propofol (Narcofol, CP Pharma) and maintained via endotracheal intubation with 1% isoflurane (Iso‐Vet, Dechra) dissolved in oxygen at a flow rate of 10 mL/kg/min.

**FIGURE 3:**
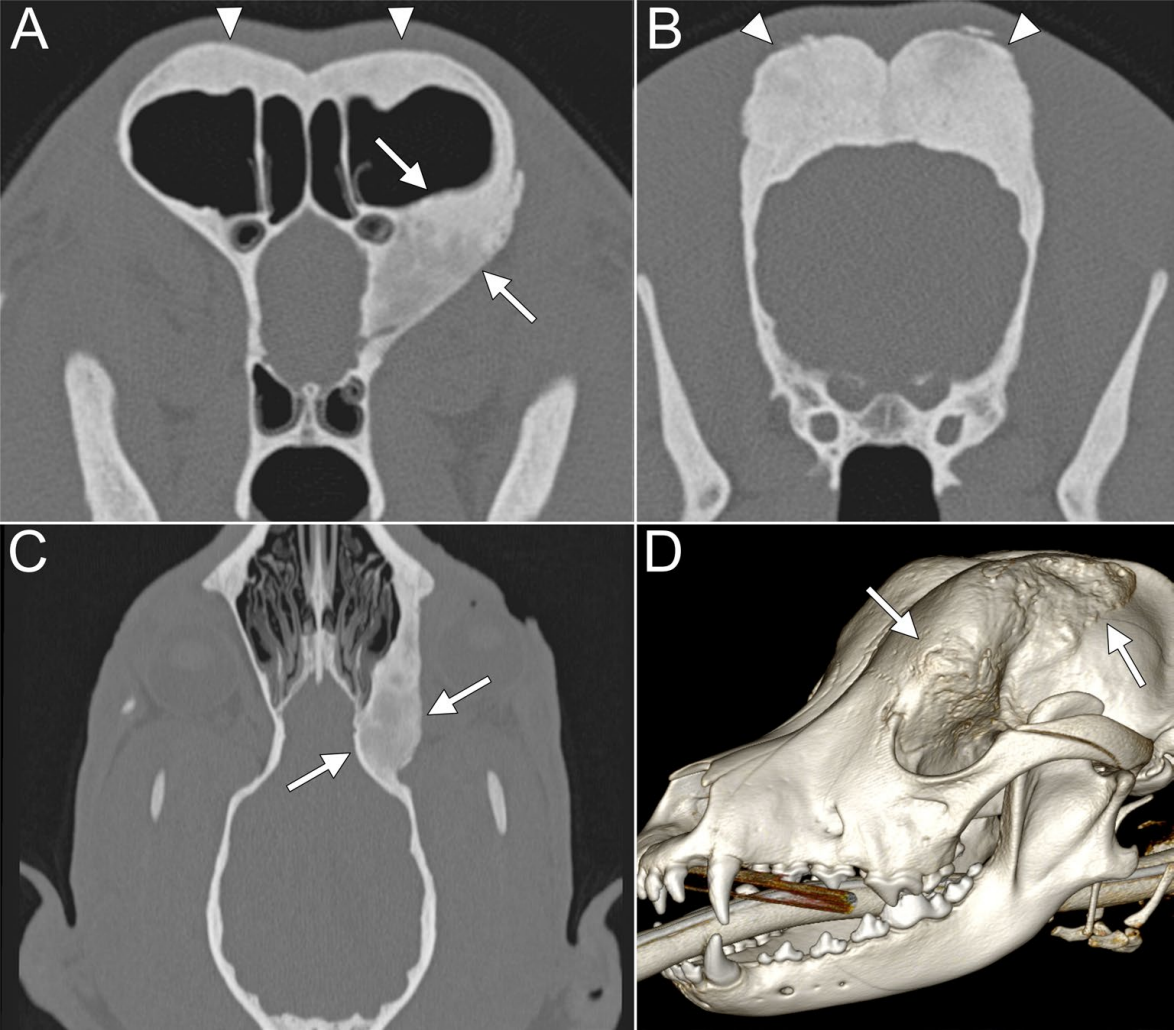
Case 1: Imaging findings. (A) Transverse CT image showing marked thickening of the orbital lamina of the left frontal bone with smoothly marginated bone proliferation and sclerosis (solid arrows), as well as moderate bilateral thickening of the squama frontalis (arrowheads). (B) Transverse CT image showing marked bilateral thickening of the parietal bone with smoothly marginated bone proliferation and sclerosis (arrowheads). (C) Dorsal CT image showing marked thickening of the orbital lamina of the left frontal bone with smoothly marginated bone proliferation and sclerosis (solid arrows). (D) 3D volume‐rendered image illustrating the extent of left frontal bone thickening and bone proliferation (solid arrows).

CT was performed for case 1 using a 6‐slice helical CT (Brilliance 6, Philips Healthcare) with a slice thickness of 1 mm, interslice spacing of—0.5 mm, pitch of 0.6, tube voltage of 120 kVp, and tube current of 150 mAs. For case 2, a 128‐slice Spectral‐CT (Philips IQon, Philips Healthcare) in helical mode with a slice thickness of 1 mm, interslice spacing of—0.5 mm, pitch of 0.389, tube voltage of 120 kVp, and tube current of 250 mAs. Scans were performed both before and after intravenous administration of 600 mg/kg of iodinated nonionic contrast medium Iomeprol (Imeron 300 mg/mL, Bracco Imaging Deutschland GmbH), delivered at a flow rate of 3 mL/s. Images were reconstructed using bone and soft tissue kernels. In case 1, CT revealed significant thickening, sclerosis, and new bone formation of the orbital lamina of the left frontal bone, along with bilateral involvement of the parietal, temporal, and occipital bones. The left frontal bone was the most affected, extending into the orbit and contributing to mild exophthalmos (Figure 3). Additionally, mild enlargement of the left mandibular, medial retropharyngeal, and parotid lymph nodes was noted. All other head structures appeared unremarkable.

**FIGURE 4:**
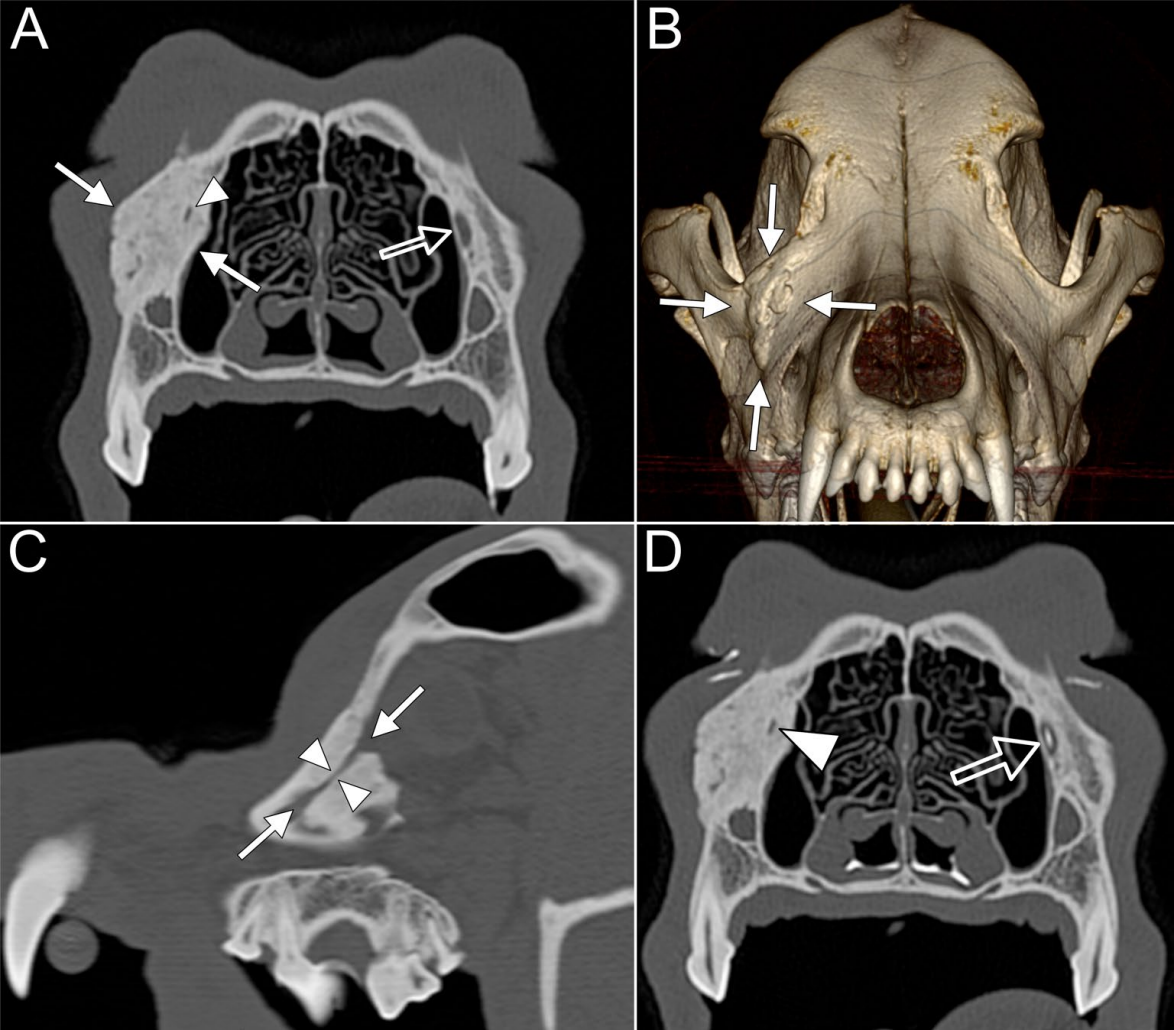
Case 2: Imaging findings. (A) Transverse CT image showing marked thickening of the right lacrimal bone with smoothly marginated bone proliferation and sclerosis (solid arrows), as well as narrowing of the right nasolacrimal duct (arrowhead) compared to the contralateral side (hollow arrow). (B) 3D volume‐rendered image illustrating the extent of right lacrimal bone thickening and bone proliferation (solid arrows). (C) Sagittal oblique CT image demonstrating narrowing of the right nasolacrimal duct (arrowheads) and the associated bone changes (solid arrows). (D) Transverse CT image following dacryocystorhinography. The narrowed right nasolacrimal duct shows no evidence of contrast filling (arrowhead), whereas the contralateral duct is filled with hyperattenuating contrast medium.

The imaging findings, along with the bilaterally symmetrical distribution of the lesions, strongly suggested CHS, with alternative differential diagnoses deemed unlikely. In case 2, CT showed focal marked thickening, sclerosis, and new bone formation of the right lacrimal bone and frontal process of the maxillary bone. This lesion extended rostrally from the medial canthus of the right eye to the level of the mesial roots of 108. The changes appeared benign, without evidence of aggressive biological behavior. The lesion expanded primarily laterally but also extended medially, encasing the nasolacrimal canal over a 1.3 cm distance, reducing its diameter to 0.56 mm compared to 1.89 mm on the contralateral side. Orthograde dacryoystorhinography (DCR) was conducted by instillation of 1 mL iodinated contrast medium Iomeprol (Imeron 300 mg/mL, Bracco Imaging Deutschland GmbH) 1:1 diluted with methylcellulose (Methocel 2%, OmniVision) into the nasolacrimal duct (NLD). Subsequent CT showed bilateral presence of contrast medium within the nasal cavity. However, no contrast medium was detected within the right NLD, whereas physiological accumulation was observed on the left side (Figure 4). The surrounding soft tissues and other head structures were unremarkable. The imaging characteristics supported a diagnosis of hyperostosis, with differential diagnosis including osteoma, osteochondroma, and exostosis. Primary malignant bone neoplasia was deemed unlikely due to the benign imaging features. Punch biopsies were obtained from the frontal bone in case 1 and the lacrimal bone in case 2.

Histopathological examination from case 1 revealed bone remodeling with sparse osteoblast activity and cortical fibrosis. In case 2, the biopsy demonstrated regularly differentiated trabecular lamellar bone with intertrabecular spaces. There was no evidence of neoplasia or osteomyelitis in either case. Additionally, bacterial culture of the sample from case 1 returned negative results.

Based on the patient histories, clinical findings, radiological characteristics, and histopathological results, CHS was strongly suspected in both cases. The condition was assessed to be in an active phase in case 1, whereas a more chronic phase was suspected in case 2.

#### 2.1.5 Outcome

The patient in case 1 was discharged with a prescription for oral amoxicillin (Amox, CP‐Pharma) at a dose of 20 mg/kg twice daily, to be continued until the microbiological results confirmed a negative finding. Additional meloxicam (Metacam, Boehringer Ingelheim GmbH) was prescribed at 0,1 mg/kg orally once daily for 1 week.

The patient in case 2 was discharged with gentamicin eye gel (Ophtogent, CP‐Pharma) to be applied locally four times daily for 5 days to prevent infection after multiple manipulations. Robenacoxib (Onsior, Elanco) was also prescribed at a dose of 1.2 mg/kg orally once daily for 1 week.

A telephone follow‐up 6 months after discharge revealed a slow regression of the left exophthalmos in case 1, with no additional complications reported by the owners.

**FIGURE 5:**
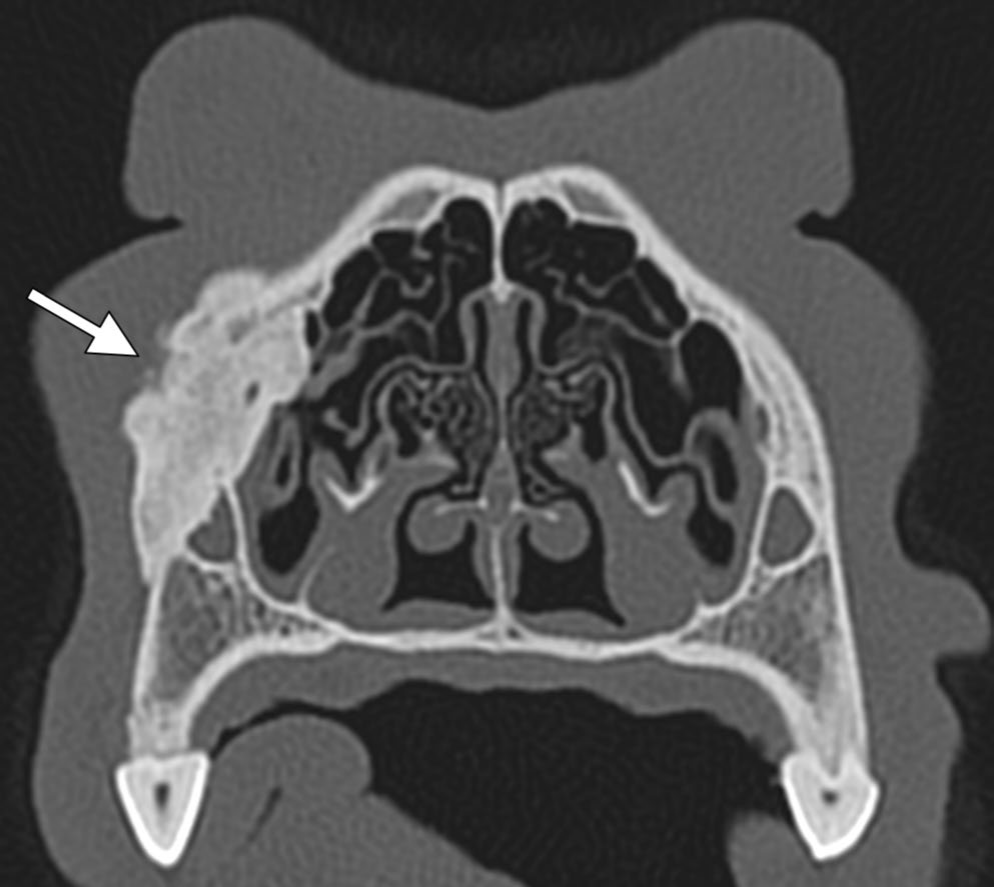
Case 2: Transverse CT image taken 4 months after the initial examination and punch biopsy. Partial consolidation of the biopsy site is observed (arrow), while the lesion remains unchanged.

In case 2, a clinical re‐examination 4 months post‐discharge indicated no changes in the clinical signs described at the initial presentation. A follow‐up CT conducted at the same time confirmed no significant progression or resolution of the lesion, though partial new bone formation was observed at the biopsy site (Figure 5).

The narrowing of the NLD in case 2 was left untreated. The owners were advised to maintain regular hygienic cleaning of the epiphora‐moistened skin and fur to prevent irritation or secondary complications.

## 3 Discussion

The term “calvarial hyperostosis syndrome” (CHS) is not uniformly defined in human literature, with various causes and associated conditions reported. It was first described in humans as a unique X‐linked recessive disorder characterized by isolated hyperostosis of the calvarium [14]. Later, CHS has been observed in humans in association with hyperparathyreoidism [15], cranial and intracranial tumors [16, 17, 18, 19], intracranial hypotension [20, 21] and seizures [22].

In dogs, CHS is characterized as an osteopathy with a nonneoplastic, proliferative, osseous lesion of the flat bones of the skull [4], which bears similarities to human infantile cortical hyperostosis (ICH) [10, 23] and craniomandibular osteopathy (CMO) [10, 24]. CHS is described as a self‐limiting osteopathy of the skull that does not involve the mandibles. Common clinical signs of the acute phase of the disease in young dogs aged 4–9 months include swelling of the skull, seizures, fever, and pain [4, 5, 6, 7, 8, 9, 10, 11, 12, 13]. The dog in case 1 of the present study exhibited clinical signs within this age range. The prolapse of the third eyelid gland may be attributable to the orbital mass, and palpation of the left skull appeared to be painful. In case 2, serous discharge began when the dog was approximately the same age as those described above; however, the specific clinical signs were absent or overlooked by the owners. Both owners have excluded a traumatic cause for the conditions observed in their dogs.

To date, exophthalmos is the only d ophthalmological effect of CHS in dogs, as seen in a young Springer spaniel [10]. This symptom was also observed in the Labrador from case 1.

In case 2, serious epiphora was the dominant clinical sign and the reason for referral. The swelling of the lacrimal bone was relatively moderate externally and went unrecognized for an extended period, which may explain the older age of the dog at the time of presentation compared to other cases.

While pain and histological signs of activity characterized by osteoblastic and osteoclastic cells define the acute phase of the disease in young dogs [9, 13], CHS is typically self‐limiting, with only mild changes persisting in most cases once the dog reaches skeletal maturity [4, 5, 6, 7, 8, 9, 10, 11, 12, 13].

These mild persisting changes were observed in the follow‐up of both cases 6 and 4 months later, respectively.

The histopathologic findings in the presented cases may represent two different stages of CHS. However, to the knowledge of the authors, clear criteria and time limits of CHS have not been established. While it is possible that the owners may have overlooked traumatic events, it seems unlikely that an injury leading to such pronounced hyperostosis—and the virtually guaranteed accompanying soft tissue reaction—would have been initially missed. Still, callus and exostosis cannot be definitively excluded. Nevertheless, the well‐organized, smoothly marginated new bone formations observed by CT in both cases, along with the relative integrity of the nasolacrimal duct in case 2, support a strong suspicion of CHS in both dogs.

A causal therapy for CHS is not known. In the two described cases, NSAIDs were administered palliatively, as for the treatment of CMO [24]. In the case of functional impairment, such as a complete obstruction of the NLD by CHS, surgical intervention would be necessary.

## 4 Conclusion

Calvarial hyperostosis syndrome (CHS) should be considered in cases of exophthalmos or lacrimal discharge, particularly when firm swelling of the flat bones is observed in young dogs.

Confirmation or exclusion of CHS should be achieved through diagnostic imaging and histological examination.

## Author Contributions

**Andrea Steinmetz:** conceptualization, data curation, investigation, visualization, writing – original draft, writing – review and editing. **Stefan Kohl:** conceptualization, data curation, resources, visualization, writing – original draft, writing – review and editing.

## Ethics Statement

Anesthesia and diagnostic procedures in the presented case were done as part of diagnosing and controlling the progression. The owners agree to the publication of the data and photographs. The local requirements have been complied with.

## Conflicts of Interest

The authors declare no conflicts of interest.

## Data Availability

The data that support the findings of this study are available from the corresponding author upon reasonable request.
